# Wearable technology in stroke rehabilitation: towards improved diagnosis and treatment of upper-limb motor impairment

**DOI:** 10.1186/s12984-019-0612-y

**Published:** 2019-11-19

**Authors:** Pablo Maceira-Elvira, Traian Popa, Anne-Christine Schmid, Friedhelm C. Hummel

**Affiliations:** 1 Defitech Chair in Clinical Neuroengineering, Center for Neuroprosthetics (CNP) and Brain Mind Institute (BMI), Swiss Federal Institute of Technology (EPFL), 9, Chemin des Mines, 1202 Geneva, Switzerland; 2 Defitech Chair in Clinical Neuroengineering, Center for Neuroprosthetics (CNP) and Brain Mind Institute (BMI), Swiss Federal Institute of Technology (EPFL Valais), Clinique Romande de Réadaptation, 1951 Sion, Switzerland; 3 Clinical Neuroscience, University of Geneva Medical School, 1202 Geneva, Switzerland

**Keywords:** Stroke, Wearable technology, Rehabilitation, Monitor, Motor function, Home-based, Remote, Telemedicine

## Abstract

Stroke is one of the main causes of long-term disability worldwide, placing a large burden on individuals and society. Rehabilitation after stroke consists of an iterative process involving assessments and specialized training, aspects often constrained by limited resources of healthcare centers. Wearable technology has the potential to objectively assess and monitor patients inside and outside clinical environments, enabling a more detailed evaluation of the impairment and allowing the individualization of rehabilitation therapies. The present review aims to provide an overview of wearable sensors used in stroke rehabilitation research, with a particular focus on the upper extremity. We summarize results obtained by current research using a variety of wearable sensors and use them to critically discuss challenges and opportunities in the ongoing effort towards reliable and accessible tools for stroke rehabilitation. Finally, suggestions concerning data acquisition and processing to guide future studies performed by clinicians and engineers alike are provided.

## Introduction

Stroke is one of the leading causes of disability worldwide [1], with a global prevalence estimated at 42.4 million in 2015 [2]. Stroke results in permanent motor disabilities in 80% of cases [3]. During the acute and subacute stages (< 6 months after stroke [4]), patients receive rehabilitation therapies at specialized healthcare centers, consisting of an iterative process involving impairment assessments, goal definition, intervention, and progress evaluation [5]. After being discharged from the rehabilitation center (i.e. after entering the chronic stage, e.g., 6 months after stroke), 65% of patients are unable to integrate affected limbs into everyday-life activities [6], showing a need for further treatment. Phrased differently, the rehabilitative process after stroke depends on the effective assessment of motor deficit and congruent allocation to treatment (diagnostics), accurate appraisal of treatment effects (recovery/adaptation evaluation), and prolonged treatment for continuous recovery during the chronic stage (extended training).

Each of these three aspects present practical challenges. Assigned treatments depend on the assessed early-stage disability [3]. A variety of assessment scales exist to evaluate motor impairment after stroke, designed to capture aspects such as joint range of motion (ROM), synergistic execution of movements, reaching and grasping capabilities, object manipulation, etc. [7]. These assessments are normally applied by specialized medical personnel, which entails certain variability between assessments [8]. Besides consistency in repeated measurements, some scales like the Fugl-Meyer assessment (FMA) [9], are unable to capture the entire spectrum of motor function in patients due to limited sensitivity or ceiling effects [10].

In addition to thorough standardized assessment scales, progress in patients is observable during the execution of activities of daily living (e.g., during occupational therapy sessions). Nevertheless, task completion not always reflects recovery, as patients often adopt different synergistic patterns to compensate for lost function [11], and such behavior is not always evident.

Main provision of rehabilitation therapies occurs at hospitals and rehabilitation centers. Evidence of enhanced recovery related to more extensive training has been found [12], but limited resources at these facilities often obstruct extended care during the chronic stage. This calls for new therapeutic options allowing patients to train intensively and extensively after leaving the treatment center, while ensuring the treatment’s quality, effectiveness and safety.

Wearable sensors used during regular assessments can reduce evaluation times and provide objective, quantifiable data on the patients’ capabilities, complementing the expert yet subjective judgement of healthcare specialists. These recordings are more objective and replicable than regular observations. They have the potential of reducing diagnostic errors affecting the choice for therapies and their eventual readjustment. Additional information (e.g., muscle activity) extracted during the execution of multiple tasks can be used to better characterize motor function in patients, allowing for finer stratification into more specific groups, which can then lead to better targeted care (i.e. personalized therapies). These devices also make it possible to acquire data unobtrusively and continuously, which enables the study of motor function while patients perform daily-life activities. Further, the prospect of remotely acquiring data shows promise in the implementation of independent rehabilitative training outside clinics, allowing patients to work more extensively towards recovery.

The objective of this review is to provide an overview of wearable sensors used in stroke rehabilitation research, with a particular focus on the upper extremity, aiming to present a roadmap for translating these technologies from “bench to bedside”. We selected articles based on their reports about tests conducted with actual stroke patients, with the exception of conductive elastomer sensors, on which extensive research exists without tests in patients. In the section “Wearable devices used in stroke patients”, we summarize results obtained by current research using a variety of wearable sensors and use them to critically discuss challenges and opportunities in the ongoing effort towards reliable and accessible tools for stroke rehabilitation. In the “Discussion” section, we present suggestions concerning data acquisition and processing, as well as opportunities arising in this field, to guide future studies performed by clinicians and engineers alike.

## Wearable devices used in stroke patients

Recent availability of ever more compact, robust and power-efficient wearable devices has presented research and development groups in academia and industry with the means of studying and monitoring activities performed by users on a daily basis.

Over the past years, multiple research groups have worked towards a reliable, objective and unobtrusive way of studying human movement. From the array of sensors and devices created, a few have gained popularity in time due to their practicality. The next subsections will focus on the wearable devices most frequently used in the study of human motion, with special emphasis on monitoring of upper limbs in stroke patients.

### Inertial measurement units (IMUs)

**Fig. 1:**
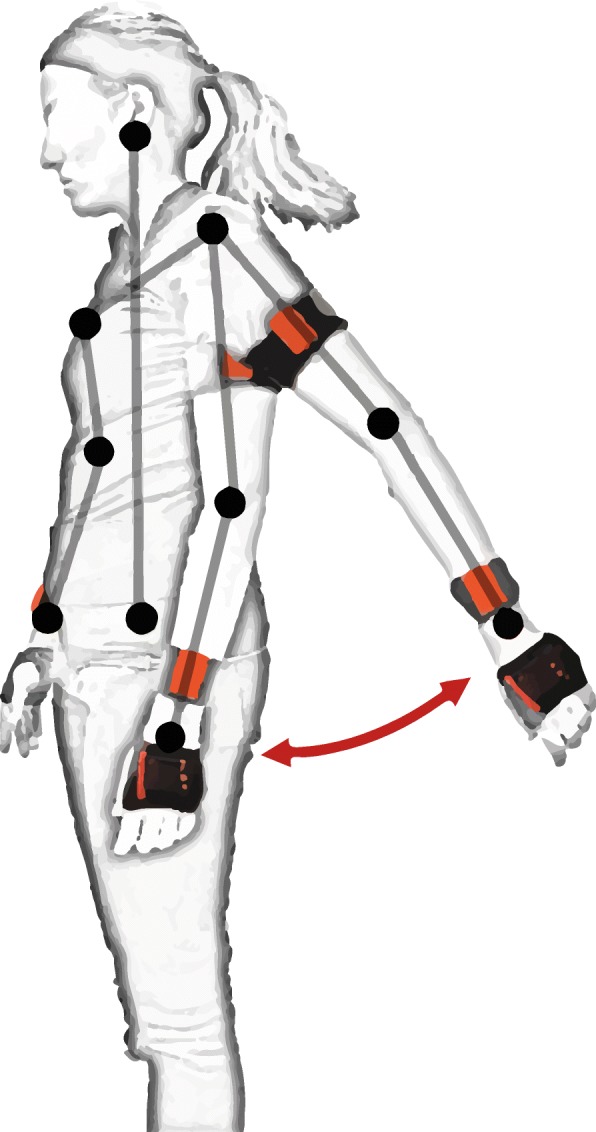
IMU sensors (orange) used to track arm movements. Sensors placed on the back of the hands, forearms and upper arms capture acceleration (linear and angular) and orientation of each segment, allowing kinematic reconstruction or movement characterization

Inertial measurement units (IMUs) are devices combining the acceleration readings from accelerometers and the angular turning rate detection of gyroscopes [13]. Recent versions of such devices are equipped with a magnetometer as well, adding an estimation of the orientation of the device with respect to the Earth’s magnetic field [14]. A general description of how inertial data are used to extract useful information from these devices is offered by Yang and Hsu [15]. High-end IMUs used for human motion tracking, such as the “MTw Awinda” sensor (Xsens®, Enscheda, Overijssel, The Netherlands) [16], acquire data at sampling rates as high as 1 kHz (sensitivities of ±2000 deg/s, ±160 m/s^2^, ±1.9 G). More affordable sensors (e.g. “MMR” (mbientlab Inc.®, San Francisco, California, USA) [17]) stream data at 100 Hz (max sensitivities of ±2000 deg/s, ±16 g, 13 G). The necessary sampling rate depends on the application, and must be defined such that aliasing is avoided (i.e. Nyquist rate, 2 times the frequency of the studied phenomenon). Figure 1 shows an example of motion tracking using these devices.

#### Diagnostics

Multiple scales exist for assessing motor function in stroke patients [7]. However, limitations exist in terms of objectivity and test responsiveness to subtle changes [18], as well as on the amount of time needed to apply these tests. Therefore, several research groups have focused on the use of IMUs to assess motor function more objectively. Hester et al. [19] were able to predict hand and arm stages of the Chedoke-McMaster clinical score, while Yu et al. [20] built Brunnstrom stage [21] classifiers, assigning each patient to one of six classes of synergistic movements in affected limbs. The Wolf Motor test [22–24], the FMA [25, 26] and the Action Research Arm Test (ARAT) [27], frequently used to assess motor function in clinical settings, have also been automated.

#### Recovery/adaptation evaluation

IMUs are practical options to assess motor function during the execution of activities of daily life. Lee and colleagues [28] focused on limb neglect and task execution quality assessment. Limb neglect can be seen by looking at the symmetry (or lack thereof) in sensor readings from the affected and unaffected sides [29–31]. Zhou et al. [32] used a single, triple-axis accelerometer to track movements of the forearm in a simple manner, but tracking of more complex motion requires either more sensors or alternative data analysis techniques. Harder-to-detect compensatory movements (e.g., of the torso) can also be identified [19]. Besides using IMU modules designed specifically for human movement tracking, interesting possibilities have been explored in every-day-use devices, such as smartphones [33].

Tracking of the whole body has also been achieved using sensor networks in an attempt to objectively evaluate movement quality in daily-life situations [34], as well as tracking of complex upper-limb movements [35].

#### Extended training

IMUs allow providing immediate feedback to patients about their performance and posture [36, 37], as well as the adequate use of equipment (e.g., orthoses) [38], which presents an opportunity for extended training (e.g., at home). Wittman and colleagues [39] used an off-the shelf system to train patients at home, seeing significant improvements as assessed by both the FMA and metrics native to the used IMU system.

#### Implementation (requirements and challenges)

The complexity of tracking and assessing motion depends on how constrained the circumstances for the recordings are. Tracking motion during the execution of daily-life activities is particularly difficult in stroke patients, as their movements are often slower, more segmented and more variable than those of healthy individuals [11]. Prolonged recordings are constrained by multiple factors, such as battery life of the wearable devices [40] and orientation drift resulting from the double integration of angular acceleration [41]. Better-performing batteries, better communication protocols (e.g., Bluetooth Low-Energy (BLE) [42]) and algorithms allowing to sample data at lower rates without losing much information (e.g., data compression [20]) help mitigate the former problem, while orientation drift can be corrected using, for example, the on-board magnetometer [41].

Recording over shorter periods, like those during standardized motor function assessment scales, is less vulnerable to these limiting factors, but still susceptible to other issues. Quantifying movements taking place in a single plane (e.g., shoulder flexion, with the arm moving parallel to the sagittal plane) is straightforward, as recordings from either the accelerometer or the gyroscope can be sufficient. In contrast, characterizing complex movements (e.g. flexor synergic movement from the FMA) is more challenging and often requires combining data from both the accelerometer and the gyroscope. Assigning clinically relevant scores (e.g. FMA scores) to performed movements requires characterizing the recorded signals using a variety of features. These features are normally extracted using a sliding-window approach along the acquired signals, and the choice of which features to use depends on the type of movements involved. Common features used in characterization of IMU data are movement intensity, signal amplitude (mean and standard deviation), signal energy and dominant frequency [43]. After extracting these features, statistical methods commonly used in machine learning allow classifying and assigning grades to the movements that originated them; the initial choice of models to test depends on the extracted features [44].

Problems can arise when studying stroke patients, as the acquired inertial signals may not hold enough information due to the very low variation of signals during slow movements. An alternative to selecting features would be to compare waveforms directly by defining a set of signals as templates for unimpaired movements with signals acquired from patients [45]. Techniques such as Coherent Point Drift (CPD) [46] or Dynamic Time Warping (DTW) [47] may be used. DTW has been used in stroke research by a number of groups (e.g. [48, 49]), as it allows to compare time series that are different in length, which is useful when comparing slower movements in stroke patients to conventional movements. CPD is a different technique for registering one set of points to another, which estimates the maximum likelihood between pairs of corresponding points and finds the best fit between them.

Sensor noise can cause huge detriment to the outcome of movement classification or assessment. The main source of noise for short-duration recordings is quantization noise (i.e., noise resulting from precision loss during analog-digital conversion), while the aforementioned drift rate plagues longer recordings [50]. Wearable sensor misplacement or misalignment can also affect classifier performance to a large extent, but some approaches have reportedly maintained precision and recall at high levels (e.g. orientation transformation, Pr. 97% and Rc. 98% [51]) during the classification of certain movements.

Table 1 provides an overview of studies using wearable sensors to study stroke patients. This table focuses on studies that included stroke patients in their cohorts.

**Table 1:** Studies involving the use of wearable sensors in the study of stroke. Only studies including actual patients shown. Most of the studies listed focused on the assessment of motor function through standardized clinical tests, which focus mainly on movement quality. This might explain the much more common use of IMU’s so far

Device Category	Sensors	Product / Units	Author	Assessment Type	Patients / Healthy controls	Methods and Results
IMU	6 accelerometers (hand, forearm, upper arm, sternum)	“Vitaport 3” (Temec®, Heerlen, NL) / m/s^2^	Hester et al., 2006 [19]	Upper limb motor assessment score prediction	12 / 0	10% relative error in prediction of clinical scores (leave-one-out cross-validation).
	6 accelerometers (hand, forearm, upper arm, sternum)	“Vitaport 3” / m/s^2^	Patel et al., 2010 [23]	FAS- WMFT	24 / 0	5.76% relative error when predicting FAS.
	1 IMU worn at the wrist	Non-commercial, / m/s^2^, deg/s	Parnandi et al., 2010 [24]	FAS- WMFT	1 / 0	“Prediction” error close to zero. *Model likely overfits the data, reasoning behind analysis might be incorrect.
	6 accelerometers (hand, forearm, upper arm, sternum)	“Vitaport 3” / m/s^2^	Del Din et al., 2011 [25]	Motor function, FM Test	24 / 0	4-points off when using a single item of the WMFT to predict total UEFM score (max 66).
	1 IMU attached to the forearm	MotionNode® (Seattle, USA) / m/s^2^, deg/s	Zhang et al., 2012 [48]	Upper limb movement trajectory comparison	2 / 1	Similarity between feature vectors for 5 exercises of the UEFM is evaluated using cosine distance. The authors claim that higher similarity (close to 0.9) corresponds to higher FM scores assigned by therapists. They compare feature vectors from affected and unaffected limbs in patients, but they never show how similar the vectors are in the healthy person.
	10 IMUs (upper and lower extremities and trunk)	Non-commercial / m/s^2^, deg/s	Strohrmann et al., 2013 [52]	Changes in motor function over time	2 / 0	Longitudinal look at changes in motor function over the course of 4 weeks. Used linear regression. Mean RMSE was of 0.15 and correlation between regression estimate and ground truth (expert assessment) was of 0.86.
	2 accelerometers, one per wrist	“GTX+” (ActiGraph®, Pensacola, USA) / m/s^2^	Bailey et al. 2015 [53]	Upper-limb bilateral activity to detect limb neglect during activities of daily life	48 / 74	Had participants wear accelerometers on each wrist for 26 h. Calculated the magnitude of the acceleration vector every second for each wrist, and a ratio of said vectors between the affected and non-affected hands. Were able to detect limb neglect in impaired patients (impairment level measured using ARAT test).
	IMU from smartphone worn on the right-front hip	“Blackberry Z10” (BlackBerry®, Waterloo, CAN)/ m/s^2^, deg/s	Capela et al., 2015 [33]	Human activity recognition (6 activities)	12 / 15	Found common features for healthy individuals (young and elderly) and stroke patients to discriminate between different conditions of movement and stillness using a smartphone. Classification accuracy was over 80% for most of the levels of comparison (e.g. mobile vs. immobile, large movements vs. stairs, etc.) when using decision trees, and similar (if slightly lower) when using SVM or Naive Bayes.
	3 IMUs (Lower arm, upper arm and trunk)	“ArmeoSenso” (Hocoma®, Volketswil, CH), “MotionPod 3” (Movea Inc.®, Pleasanton, USA) / m/s^2^, deg/s, Gauss	Wittman et al. 2016 [39]	Home-based rehabilitative training	11 / 0	Significant improvement of motor function as assessed by the FMA (4.1 points) and by metrics native to the “ArmeoSenso” system
	2 accelerometers (forearm and upper arm)	Not specified / m/s^2^	Yu et al.,2016a [20]	Brunnstrom stage classifier	23 / 4	Used ELM to classify people into 5 of the 6 stages of the Brunnstrom Stage Evaluation. 80% of samples were used as training set. No cross-validation was done. All patients belonged to stages from II to V. Stage VI is considered to be unimpaired, so data acquired from healthy participants were used. Data were acquired during a single exercise (repeated several times) and used to predict Brunnstrom stage. Accuracy was above 85% when using ELM.
	1 IMU worn at the forearm	“MTi-300” (Xsens®, Ensched, NL) / m/s^2^, deg/s	Zhang et al., 2016a [35]	Upper limb motion classification	14 / 0	Recorded inertial data from 14 patients (6 were relatively unimpaired). Used PCA and used top 7 components to label recordings according to the motion that generated them. The Fuzzy Kernel algorithm achieved an error rate of 0% when dealing with the 6 well-recovered patients, and of 0.56% for more impaired patients.
	1 IMU worn at the forearm	“MPU-6050” (InvenSense®, San Jose, USA) / m/s^2^	Zhang et al., 2016b [49]	Upper limb motion assessment	21 / 8	Proposed a mobility index based on DTW to characterize patients’ movements and assign them to Brunnstrom stages from III-VI. Their index used with a KNN classification algorithm (k = 3) achieved an accuracy of 82% in leave-one-out cross validation.
	2 accelerometers, one per wrist	“LSM9DS0” (Adafruit®, New York, USA)/ m/s^2^	de Lucena et al., 2017 [30]	Bimanual symmetry, jerk and clinical function to explain variance in upper limb recovery	9 / 0	Used PCA and concluded that the first component relates to functional status, whereas they suggest the second component might be related to movement quality (as it described a strong correlation (≥0.75) between acceleration asymmetry and jerk asymmetry). Both principal components were found to explain 86% of the variance.
	1 IMU worn at the wrist	“ReSense” [54] / m/s^2^, deg/s	Leuenberger et al., 2017 [29]	Affected limb neglect during activities of daily life	10 / 0	Proposed a new measure for arm use called Gross Arm Movements, which detects changes in arm orientation larger than 30 degrees. This measure has large correlation to clinical tests (*r* > = 0.9) even when not removing signals acquired while patients walk.
	2 IMUs, one per wrist	“Shimmer3” (Shimmer Research®, Ireland) / m/s^2^, deg/s	Lee et al., 2018 [28]	Neglect and exercise quality at home	20 / 10	Detection accuracy of goal-directed movements was described with a ROC curve, with an AUC of 87%. F-score (harmonic mean of precision and recall) of 84.3% when classifying movements into feedback vs no-feedback groups, an F-score of 73.7% when detecting feedback due to accuracy issues and an F-score of 65.3% when detecting feedback due to compensatory movement.
EMG	10 EMG electrodes on forearm and hand	Noraxon® (Scottsdale, USA) / mV	Lee et al., 2011 [55]	Classification of hand postures (6 classes)	20 / 0	LDA to classify signals into 6 hand gestures, with accuracies ranging from 37.9% (severely impaired subjects, Chedoke stage 2 and 3) to 71.3 (moderately impaired, Chedoke stage 4 and 5). Single model built for each patient, gradually adding more data to it.
	89 EMG electrodes	“Refa 128” (TMSI®, Twente, NL) / mV	Zhang & Zhou, 2012 [56]	Classification of hand postures (20 classes)	12 / 0	Used Fisher linear discriminant analysis (PCA + LDA) for dimensionality reduction. Best performance (96% classification accuracy) was obtained using time-domain features and an SVM classifier. Achieved comparable results with only 8 electrodes, but do not specify which ones
	2 EMG electrodes	Not specified / mV	Donoso Brown et al., 2015 [57]	Home-based gamified, rehabilitative training	10 / 0	Proved feasibility of this approach at home, and the system was described as engaging and motivating, but there were no reports of improved functionality transferred to activities of daily life
Pots. & Encoders	2 potentiometers	“SP12S-1 K” (ETI Systems®, Carlsbad, USA) / V mapped to angular displacement	Durfee et al., 2009 [58]	Hand joint angles tracked during proof of concept rehabilitative game	24 / 0	System of beams tracking wrist and index finger joint angles. No classification or other form of accuracy reported, as position was mapped directly from potentiometer readout.
	1 encoder	“E4” (US Digital®, Vancouver, USA) / V mapped to angular displacement	Chen et al., 2017 [59]	Hand joint angles in 4-bar hand orthosis	10 / 0	Patients trained at home (only 7 finished training) during 4 weeks, training 5 times a week. By the end of training, patients showed motor improvement of 4.9 +/− 4.1 points in FM score, with a strong correlation (0.90) between amount of movements performed during training and score improvement.
Flexible sensors	Flex sensors along the dorsal side of fingers	“Flexpoint bend sensor” (Flexpoint Sensor Systems®, Draper, USA) / V mapped to joint flexion	Prange-Lasonder et al., 2017 [60]	Hand gesture tracking during rehabilitative training and assistive grasping	5 / 0	Presented a glove with two possible modalities (assistive and rehabilitative). Modest improvement in pinch force and execution of other tasks was reported, hinting towards potential benefits of its use as a rehabilitative/assistive tool.
Combinations	2 accelerometers (forearm and upper arm) and 7 flex sensors (dorsal side of fingers, wrist)	“ADXL345” (Analog Devices Inc.®, Norwood, USA) / m/s^2^, V mapped to joint flexion	Yu et al., 2016b [61]	Motor function, FM Test	24 / 0	Evaluated shortened version of UEFM using accelerometers and flex sensors. Model built using SVM (after using RRelief algorithm for feature selection) had a 0.92 correlation with clinical scores given by a therapist.
	2 IMUs (wrist, upper arm) and 10 sEMG (forearm, biceps and triceps)	“MPU-9250” (InvenSense®, San Jose, USA), EMG not stated / m/s2, deg/s, mV	Li et al., 2017 [62]	Motor function, FM Test	18 / 16	34-leave-one-out cross validation, achieved determination coefficients (correlation between their proposed measure and FM score) of 0.85 using different unsupervised (PCA, NMF) and supervised (LASSO) algorithms.
	8 EMG + IMU, worn at the forearm	“Myo” armband (Thalmic Labs®, Kitchener, Canada) / m/s^2^, deg/s, arbitrary units (Myo EMG)	Ryser et al., 2017 [63]	Control signals for a hand orthosis	3 / 0	Classification accuracy between 78 and 98% in stroke patients when discriminating between 3 hand gestures using SVM.
	9 IMUs (hands, wrists, upper arms, forearms, sternum) and 16 EMG electrodes (worn at the forearms)	“Myo” armband, IMU not stated / m/s^2^, deg/s, arbitrary units (Myo EMG)	Repnik et al., 2018 [27]	ARAT test	28 / 12	Correlation of 0.60 between movement time and movement smoothness with respect to ARAT score. EMG data revealed significant differences in muscle activity of healthy subjects when grasping objects of different sizes. Normalized muscle activation revealed that, in more affected patients (ARAT score 2), maximal muscle activation was present when grasping the largest object, while in healthy participants activation was close to one third of maximal output.

### Surface electromyography (sEMG)

**Fig. 2:**
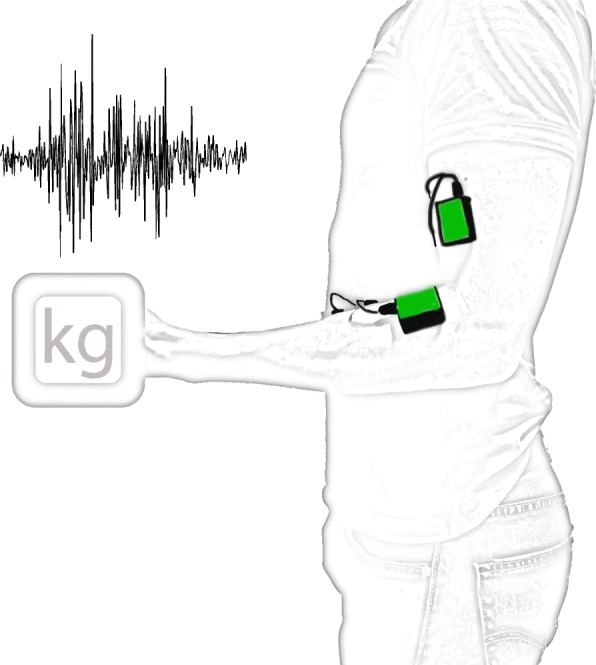
EMG sensors (green) placed over biceps and flexor digitorum superficialis muscles, involved in elbow and wrist flexion, respectively. Electrodes placed asymmetrically with respect to the neuromuscular plaques allow capturing the electrical potential difference as the depolarization wave travels along the muscle cells’ membranes. Resulting signal (top left) is filtered and amplified for further processing

Surface Electromyography (sEMG) is a technique in which the electrical potential generated whenever muscles contract is measured using electrode pairs placed on the skin over the muscles. The electrodes need to be asymmetrically placed with respect to the neuromuscular plaques in order to capture the electrical potential difference as the depolarization wave travels along the muscle cells’ membranes. Figure 2 shows a typical placement configuration for EMG devices, intended to record activity from contracting muscles involved in elbow and wrist flexion. Effectively capturing all significant frequency components of the EMG signal (according to the Nyquist rate) requires a sampling rate of 1000 Hz, as its highest frequency components are reportedly around 400–500 Hz [64]. Still, frequencies needed depend on the circumstances of the recording and its corresponding analysis. For instance, Ives and Wigglesworth [64] showed significant decreases in amplitude (11.4%) and timing (39 ms signal lengthening) when comparing a sampling rate of 6 kHz to 250 Hz. These differences would likely not affect the performance of a classifier if all data were recorded with the same sampling rate, but might impede classification if sampling rates were too different because of different amplitudes and timing shifts. High-end acquisition systems, such as “Ultium” wearable EMG sensors (Noraxon Inc.®, Scottsdale, Arizona, USA) [65], have sampling rates as high as 4 kHz (sensitivity of 0.3 μV in a range of 0–5 V), while more accessible alternatives like the “FreeEMG” (BTS Bioengineering®, Garbagnate Milanese, Milan, Italy) [66] have a sampling rate of 1 kHz.

#### Diagnostics

Wearable EMG sensors have high potential in the study of stroke patients. Investigation of neural activity as measured through motor-evoked potentials (MEPs) triggered by Transcranial Magnetic Stimulation (TMS) [67] is simpler with wireless EMG. EMG sensors can complement inertial data from IMUs during standardized motor function assessments. For example, Li and colleagues [62] improved the correlation in 0.5% between their condensed measure of motor function and the FM score assigned by a clinician. Albeit the modest increase, assessment of dexterous movements, grasping exercises and applied force is not practical with IMUs, but can be characterized with selected EMG features (e.g. area under the curve correlating with applied force), which argues in favor of including this sensor type during motor assessments. Repnik and colleagues [27] complemented IMU data with EMG during the assessment of the ARAT test to capture dexterous movements involved in the manipulation of small objects, finding significant differences in muscle activation of healthy subjects according to the size of grasped objects, and similar (maximal) muscle activation in more impaired patients (ARAT score 2) when grasping the largest object.

#### Recovery/adaptation evaluation

After stroke, patients tend to adopt compensatory strategies to accomplish motor tasks, especially in case of moderate to severe impairment [11]. These compensatory behavior might go unnoticed during a regular assessment, but can be captured and quantified using recordings from EMG sensors [68].

#### Extended training

Wearable EMG sensors allow providing online feedback during home-based training in a similar way as with IMUs. Instead of tracking gross arm movements, applied force calculated from recordings of muscle activity can serve as a parameter to provide feedback during training. EMG-based biofeedback has been reported to lead to enhanced motor improvements [69], and Donoso Brown and colleagues [57] used it to test a gamified form of home-based training, although they did not find any improved functionality derived from their intervention.

#### Implementation (requirements and challenges)

After amplification and preprocessing (e.g. signal filtering for de-noising), these signals can be used to identify patterns of activation related to specific movements or postures. The type of processing applied to recorded signals depends on the application. For example, continuous recordings of muscle activity during the execution of activities of daily living requires epoching the signals, keeping only relevant segments capturing discrete events of interest. It is possible to do this segmentation manually, but automated methods of threshold detection are a much more practical option [70]. After removing signal segments deemed irrelevant, an adequate processing pipeline must be implemented depending on the information sought. Extracting information about motor-unit activity while performing e.g. activities of daily living is possible through wavelet analysis or a variety of time-frequency approaches [70]. In contrast, identification of gross arm movements and hand gestures, as well as their assessment during motor assessments, is often approached by extracting meaningful features out of a sliding window. Some groups tried correlating their own measures to scale scores without a formal validation of their measure, which makes interpretation difficult and supports an approach of direct label/score prediction in the context of standardized tests.

As described for IMUs, a sliding-window approach allows extracting significant features for later classification. Classification is generally performed using signal features (i.e. root mean-square, amplitude, etc.) [71] chosen based on the type of movements in question. Alternatively, extracting many features and applying feature selection criteria afterwards [72] is also possible.

Classification accuracy tends to be high when only a few (five or six) classes (each corresponding to a gesture to be identified) are involved, but accuracy frequently decreases as more gestures are added. Further detriment to classification performance occurs when dealing with highly impaired stroke patients, as their muscle signals tend to be less pronounced [55]. Electrode number and distribution plays a role as well; high density EMG, with over 80 electrodes placed as a grid on the upper arm, forearm and hand, has yielded high classification accuracies when dealing with many hand postures, but the use of only a few well-placed electrodes yields comparable results [56]. Arrays of electrodes placed on the forearm offer a good tradeoff between relatively simple setups and useful data acquisition leading to acceptable classification accuracies. Pizzolato et al. [73] compared an inexpensive device, consisting of eight single differential electrodes worn as a bracelet, to more complex and much more expensive systems. They reported a reasonably high classification accuracy (69.04% +/− 7.77%) with a setup of two adjacent bracelets (16 electrodes).

There are several factors affecting the EMG signal. Repeated recordings performed on the same test subjects during several days has been reported to decrease hand-gesture classification in close to 30%, compared to results obtained from repeated measurements taking place during the same day [74]. This might result from sensors being placed in slightly different locations, as altering the position of an electrode by just one centimeter can result in amplitude variations of 200% [75]. Hermens and colleagues offer a series of recommendations on sensor placement and orientation to decrease this variability [76].

Other sources of EMG noise affecting the performance of used classifiers include cable motion artifacts, power-line noise, thermal noise from the sensor’s electronic components, electrochemical noise from the interface between the electrodes and the skin and mechanical disturbances [70]. Currently-available wearable EMG sensors are mostly affected by mechanical disturbances, which can be filtered out by applying a high pass filter with cutoff frequency at 20 Hz [77]. The choice for applied filtering also depends on the application. For example, low frequencies (i.e. 1–5 Hz) contain important information for hand gesture classification [78], which would be filtered out with the 20 Hz high-pass filter.

### Potentiometers and encoders

**Fig. 3:**
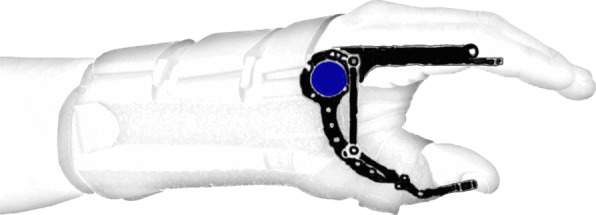
Encoder (blue) mounted on a hand orthosis, aligned with the rotation axis of the index finger. This configuration allows tracking angular displacement of fingers supported by the orthosis

An accurate way of measuring the angular displacement around joints is by means of potentiometers and encoders. Potentiometers are devices containing a conductive disc with a certain resistance and two contact points on top. The distance between these contact points can vary, which results in more or less resistive material between the contact points. As resistance varies in an approximately linear way with changes in arc length, it is possible to map a direct relationship between resistance and angular displacement. This means that aligning the knob to the rotation axis of a joint allows a good estimation of its angular position. Encoders are optical sensors containing a slitted disc. A LED (light-emitting diode) shines against the disc, which allows light to pass through the slits but blocks it otherwise. Presence and absence of light, detected by a photosensitive component, is encoded into ones and zeroes and is used to determine angular displacement. Potentiometers are analog sensors with “infinite” resolution, whereas encoders can have resolutions as high as 1 million counts per revolution [79]. Figure 3 shows an encoder mounted on a hand orthosis to track the fingers’ angular position.

#### Diagnostics

Encoders and potentiometers can be used in clinical environments to measure ROM in patients. Researchers at Peter S. Lum’s lab [80, 81] built an orthosis consisting of four bars coordinating the movement of the metacarpophalangeal finger joints and the thumb metacarpophalangeal joint for home-based training in stroke patients, using encoders to calculate the joint angles.

#### Recovery/adaptation evaluation

Chen and Lum [82] focused on an “assists as needed” approach, using a combination of potentiometers and encoders to calculate the joint angles of an arm exoskeleton and using this parameter to adjust therapeutic training. Lim et al. [83] combined accelerometers with a different encoder using a slitted strip instead of a slitted disc. This sensor detects the linear displacement of the strip, which means that laying the strips along the links of interest (i.e. fingers) allows the measurement of joint angles without aligning the rotation axes, facilitating its use during the execution of daily life activities.

#### Extended training

Chen and colleagues [59] studied the effects of training with an encoder-equipped hand orthosis at home, finding significant improvements in FMA score (4.9 ± 4.1 points).

#### Implementation (requirements and challenges)

The advantage of not needing to apply machine learning algorithms notwithstanding, the need of a parallel structure (e.g., exoskeleton) or embedding them in a glove restricts the range of applications these sensors may have for stroke patients. Donning and doffing equipment might be challenging for patients with low dexterity or high spasticity [60].

### Conductive elastomer (CE) and other flexible sensors

**Fig. 4:**
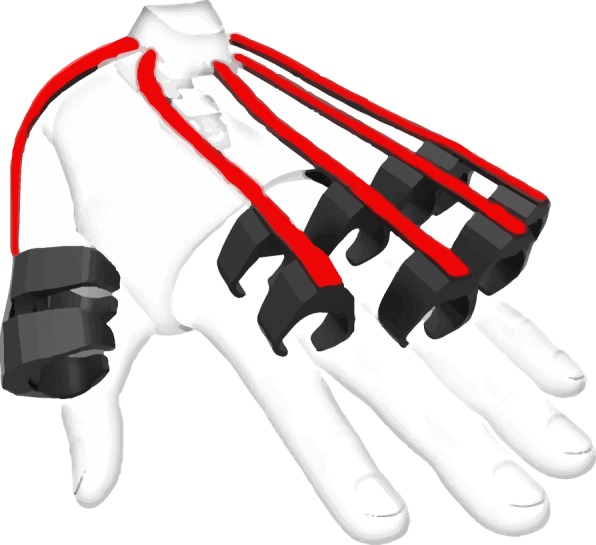
Flexible sensors (red) laid along the fingers. Their flexion results in piezo-resistive changes in the conducting material (e.g. silver nanoparticles), which map directly to different finger positions. Prototype IMU sensor glove by Noitom [84]

Conductive Elastomer (CE) sensors are flexible components with varying piezo-resistivity. Piezo-resistivity changes due to deformations suffered by a textile substrate deposited with conductive particles (e.g. silver nanoparticles). When placed along a moving body part, such as fingers, it is possible to map the sensor readout related to a particular deformation of joint angles. Figure 4 shows an example of flexible sensors tracking the position of individual finger movements.

#### Diagnostics

Yu and colleagues used flexible sensors in combination with IMUs to assess motor function [61], and obtained results bearing a high correlation (0.92) with clinical scores given by a therapist. Flex sensors are frequently used as “gold standard” when attempting measurements with others setups (e.g. [85]).

#### Recovery/adaptation evaluation

Movement tracking using deformable sensors embedded into clothes would allow monitoring patients as they perform activities of daily living. For example, Tognetti et al. [86] embedded CE sensors into clothing with the objective of classifying body postures and hand gestures (with a reported sensitivity of 11,950 Ω/mm), a work further developed by Giorgino et al. [87, 88] and De Rossi [89]. A more complex system, combining this technology with EMG and IMU data was presented by Lorussi et al. [90]. The use of piezo-resistive fabric [91] and fabric-based microelectromechanical systems (MEMS) [92] offer alternatives to CE sensors. All these studies show promise in the use of flexible sensors embedded in clothing to monitor stroke patients, but testing with stroke patients is still lacking.

#### Extended training

Prange-Lasonder and colleagues [60] implemented a gamified form of a rehabilitative training using a glove equipped with flexible sensors, and studied the effects of such training at home [93]. Their results proved the feasibility of this approach as a home-based therapy, even though they did not find significant differences in comparison to their control intervention.

#### Implementation (requirements and challenges)

Flexible sensors embedded into clothing constitute an attractive option for unobtrusively tracking movements in stroke patients during motor assessments, execution of daily living activities, and rehabilitative training. At present, their use in clinical environments and in-home settings is difficult due to practical issues related to donning, doffing and washing the garments. Furthermore, some sensors require a large amount of wiring [91], which reduces the degree of unobtrusiveness. Additionally, mechanical deformations resulting from, for example, wrinkles in the fabric [88] introduce noise to the system, complicating posture and movement tracking.

## Discussion

Stroke is a frequent disorder that often results in long-lasting loss of motor functions. After stroke, the rehabilitative process relies on three main elements: 1. Diagnosis, in which clinicians use standardized scales to estimate maximum recovery for every patient [94] and assign them to rehabilitation therapies accordingly [95]. 2. Evaluation of recovery or adaptation, during which clinicians assess the extent up to which patients can perform activities of daily living. 3. Extended training, necessary for patients with persistent motor impairment after entering the chronic stage.

Conventional motor assessment is vulnerable to biases derived from measurement errors [96] and ceiling effects [97], whereas compensatory strategies frequently adopted by patients while performing different tasks [11] can complicate the appraisal of recovery. Therapy and training provision at healthcare centers is limited to available resources and restricted by its corresponding costs, which obstructs prolonged rehabilitative training for patients who do not recover fully within the first months after stroke.

A promising option to assess stroke patients objectively resides in the use of wearable technology. As high-end sensors become more accessible, more reliable and less obtrusive, the chance of acquiring relevant data during patients’ training or daily routines gets easier. A variety of wearable sensors (e.g. [29, 49, 59, 60, 62, 98]) have been used to assess several aspects of motor performance in stroke patients, going from motor impairment to more subtle forms of behavior, such as limb neglect.

In the present paper, we seek to compare different setups with the intention of finding the most promising candidates for different applications. There are four main wearable sensors used in the study of stroke: IMUs, EMG, potentiometers/encoders and flexible sensors. IMUs allow measuring changes in acceleration, inclination and orientation unobtrusively. Wireless, energy-efficient [42] transmission of data characterizing these sensors enables whole-body recordings through sensor networks [34], supporting this sensors’ candidacy for movement tracking [28, 35, 49]. Several groups have used IMUs with diagnostic purposes [19–27] and to assess the execution of daily-life activities [19, 28–33]. High portability and accessible costs further support these sensors as an option for prolonged training during the chronic stage (e.g. at home) [39]. There are general complications inherent to the use of these devices, such as estimation errors derived from accumulated error in the calculation of orientation from angular acceleration (i.e. orientation drift [41]) and quantization noise [50]. In addition, high movement variability in stroke patients, resulting from adopted compensatory muscle synergies and slower, segmented movements [11], complicate data characterization and comparison.

EMG wearable sensors have also been used for diagnosis [27, 62] and first attempts at extended training outside clinical environments [57]. Monitoring the execution of activities of daily living can benefit from EMG recordings, as these sensors allow capturing differences in muscle pattern activations resulting from compensatory movements [68]. These sensors can complement the information obtained with IMUs. Aspects neglected by some assessment scales (e.g. FMA), such as applied force [10], can be derived from muscle activation as recorded with EMG. EMG sensors are susceptible to different sources of noise, which must be removed before signals can be used [70]. Furthermore, variable placement of electrodes can also mislead estimations and affect the performance of the models used to classify measured activity.

Potentiometers and encoders are robust to noise and require little processing of signals, as the output from these sensors can be mapped directly to angular displacement (or linear, in the case of linear encoders). The range of applications in stroke for these sensors is limited to measuring ROM of limbs, and requires mounting them on a parallel structure, such as an orthosis, limiting the degrees of freedom of measured movements. Still, their potential in extensive home-based training is clear [59]. The need for an orthosis disappears with the use of linear encoders [83] due to integration of the sensors into gloves. Nevertheless, the use of both orthoses and gloves can be difficult for patients suffering from hand spasticity, which would complicate their use at home. This problem persists whenever using flexible sensors embedded in gloves. Flexible sensors embedded in clothing could be a viable option for tracking everyday life activities, but practical issues related to washing the garments and to the large amount of wiring required still impede their regular use.

As IMU and EMG data cannot be mapped directly into the movements and actions that generated them, acquired signals must be processed differently. Depending on the objective (e.g. assign grades to movements, compare patients to healthy controls, etc.) data can either be classified using different forms of statistical processing, such as common methods applied in machine learning [99], or compared using algorithms like DTW [48, 49]. Built models often fail to generalize to data from highly impaired patients due to lower signal-to-noise ratio (SNR) [55]. Further, results are hard to compare due to a lack of a unified data acquisition protocol [73].

### Choosing an adequate setup

The choice for the best setup depends on the intended application. The best candidate to study movement quality while remaining unobtrusive and easy to deploy is likely IMUs. Data from IMUs provide enough information to characterize movement execution (e.g. [49]), detect limb neglect and assess performance of activities of daily life [28]. During motor assessments, overlooked functional information (e.g. muscle activity) [10] can be acquired using EMG [62]. The best candidate to identify hand gestures (e.g. for orthotic control) amongst the sensors discussed here is likely EMG. EMG allows identifying hand gestures effectively without altering too much the way in which patients interact with the environment, as would be the case with potentiometers and flexible sensors. A possible alternative would be the use of pressure sensors [100]; Sadarangani and colleagues [98] tried this approach with stroke patients and achieved classification accuracies above 90% (3 classes only). We excluded this type of sensor from the present review because there is, to the best of our knowledge, no wearable version yet.

### Data processing: recommendations

As mentioned earlier, the analyses pipeline depends heavily on the object of study (e.g. movement quality, limb neglect, etc.). There are multiple features to characterize EMG and IMU signals for later classification (e.g. into classes related to motor function), and the choice depends on the property of interest. For example, muscle force is well- represented using the RMS of the EMG signal, whereas movement quality can be better observed by calculating jerk (rate of change in acceleration, capturing movement smoothness) from IMU data. Alternatively, comparing waveforms directly requires either normalizing the length of the time series or somehow matching them to account for different signal durations, such as with DTW.

For classification problems, it might be better to have many features and then trim them down by means of PCA or other relevance determination algorithms (e.g. RRelief). This is a necessary step, as dataset sizes are often quite small, and keeping too many features might result in models not generalizing to new data (overfitting). The choice for the model depends on the application and on its final objective. Several studies discussed in Table 1 used SVM in classification, and some of them reported testing more than one model, but this choice is not compulsory. For example, if the objective is to deploy an automated tool for assessment of motor function and the ultimate goal is for it to reliably assess functionality, many different models can be tested and optimized to find the best performer. Alternatively, applications such as allocating patients to different therapies based of their specific needs (i.e. individualized care) might benefit from transparent, easily explained models such as decision trees, as the rationale behind a choice for therapy is important.

The way in which models are fine-tuned and validated is an important aspect too. Several studies shown in Table 1 claim performing cross-validation, but its actual implementation varies a lot between studies. A good approach is to separate a portion of the data as test data and leave it “untouched” until after fine-tuning the model using the remaining data (i.e. training data). Once more, a fraction of these data is set aside, this time as validation data, while using the rest to fit the model. Repeating this process with the training data and averaging (or “voting”, i.e. selecting most frequent labels) the results will yield a less-biased model. Subsampling of data for every iteration can be done with replacement (bagging) or without (pasting). After fine-tuning the model’s parameters, plugging-in the test data gives a more realistic impression of how well the model will generalize to new data. In the end, results obtained will depend on the quality of used features and on the amount of information contained in them. The optimization of the models is relatively trivial, in the sense that there are many available tools to do so. Time and effort must be invested in feature engineering, as models can only perform as well as the quality of the information used to build them.

In general, the more data is available to train models, the better. The most effective algorithms used across domains, such as neural networks, are only useful if used on large amounts of data. For this reason, initiatives like the “NinaPro” database [101] should be supported and contributed-to, such that data acquired on different sites might be pooled together. Data acquisition and sharing between different sites brings along its own challenges and escapes the scope of this review, but standardized protocols like the “NinaPro” and guidelines for sensor placement (e.g. [76]) will be crucial towards this effort.

### An empty niche

An EMG + IMU device that had been gaining momentum in multiple scientific domains was the “Myo” armband (Thalmic Labs®, Kitchener, Ontario, Canada) [102]. This device consists of an array of eight single differential electrodes and a 9-axis IMU, presented as a bracelet, transmitting data through BLE. Its affordability and user-friendliness made it an attractive alternative for prolonged, possibly unsupervised recordings. Furthermore, a formal comparison between this armband and several high-end EMG systems showed similar classification accuracies when using two armbands at the same time [73] to classify signals into 40 different movements, further supporting the use of this device in research. Applications for motor assessments [27], orthotic [63] and prosthetic [103] control, gesture recognition [104], etc. have benefited from this device. CTRL-Labs® (New York City, New York, USA) [105] is developing a new device combining these sensors, but this important niche is, at present, unattended. Some institutions in China have started selling products significantly inspired by the “Myo”, such as OYMotion® (Beijing, China) [106], but their acquisition in Europe and America can be problematic, prices are high, and there are no reports on how well they perform.

### Alternatives and possibilities

Easily deployed, inexpensive IMU devices are available off-the-shelf. Mbientlab [17], for example, offers a wide array of what seems to be modular and flexible IMU setups allowing prolonged recordings with multiple sensors simultaneously. Beange and colleagues [107] compared one of the IMU modules to a motion capture system and found its performance acceptable. High- end systems such as the Xsens [14] perform excellently, but their prohibitive cost limits the range of possible applications; such a system could only be used for measurements in high-end, specialized clinics, failing to solve the problem of limited resources of common healthcare centers.

As for the acquisition of EMG data, we were not able to find a low-cost solution providing quality data while remaining simple to use. Systems built by companies like Noraxon [65], Delsys® (Natick, Massachusetts, USA) [108] or Cometa® (Bareggio, Milan, Italy) [109] provide high quality data, but at a high cost. Less expensive systems like “FreeEMG” [66] or “Biometrics’ sEMG sensors” (Biometrics Ltd.®, Newport, UK) [110] are more accessible, but are still suboptimal in the sense of requiring careful placement of gel electrodes, which makes it impractical for unsupervised patient use at home.

Presenting a similar design to that of the “Myo” armband, Yang and colleagues [111] built a bracelet equipped with textile electrodes, reporting high classification accuracy (close to 100%) in hold-out cross-validation. The study involved only three healthy participants, and training and testing data used in cross-validation came from the same subject (no inter-subject validation). Still, the design of this device seems promising.

A different approach trying to enhance EMG systems with near-infrared spectroscopy (NIRS) was taken initially by Herrmann and Buchenrieder [112] in an attempt to reduce electrode crosstalk. This approach was also pursued by a couple other groups [113, 114], but challenges related to the time resolution of NIRS limit the applications possible for these devices.

Interesting possibilities exist in the realm of printable (i.e. epidermal electrodes [115]) and temporary tattoo electrodes [116], but these are not yet readily available for deployment. For the time being, the choice of a device to acquire inertial and EMG data simultaneously in an inexpensive, easy to deploy fashion remains an open question.

### From bench to bedside

#### Wearable sensors in clinical environments

The processing steps and the implementation challenges described before may appear daunting when thinking about integrating these sensors into clinical practice. The importance of discussing these challenges lies in the joint effort towards democratizing these technologies such that their advantages might be widespread, accessible to all, their performance and reliability ensured. To achieve this goal further research is necessary, and research can greatly benefit from knowledge acquired in the clinic.

There is a variety of readily available systems dedicating wearable sensors to rehabilitation. For example, the “ArmeoSenso” system (Hocoma®, Volketswil, Zürich, Switzerland) [117] uses IMU’s alongside a gamified form of training (this is the system used for home-based training, mentioned before [39]). For EMG, products like Cometa’s “EMG Easy Report” [118] or Noraxon’s “myoMuscle” [119] allow simplified analyses, like pairing recordings to video, to look at muscle activity related to specific movements. The use of these systems in the clinic provides further insights into practical aspects to consider when developing new products, and allows fitting these technologies to the patients’ needs. Their functionality may be limited to certain aspects and system errors might display these techniques as less efficient than conventional approaches, but the development of flexible and robust systems requires this sort of iterative testing in real-life situations, enriched with the knowledge of specialized medical personnel. Even if the transition towards the integration of these devices into clinical practice represents an extra effort on an already strained environment, it has potential at reducing costs once they become ubiquitous.

Hughes and colleagues [120] reported that one of the main obstacles in the way of adopting these type of technologies in clinics is the lack of awareness about their existence, which calls for better communication and collaboration between researchers and clinicians.

#### The international classification of functioning, disability and health (ICF)

The ICF is an important and well-established tool in clinical neuro-rehabilitation and seeks to provide a framework based on two models of disability, one coming from individual factors and another from social factors [121]. This biopsychosocial model provides standardized grounds for studying, understanding and addressing disability. Metcalf and colleagues [122] assessed which of the most frequently used scales of motor function in stroke patients better fit the framework of the ICF in terms of repeatability and reliability, rating as most reliable those test involving numerical assessments such as ROM and movement time. Using wearable sensors during regular assessments will then improve performance of standardized motor assessments in the framework of the ICF.

Escorpizo and colleagues [123] proposed two main actions towards the integration of the ICF into clinical practice, one of which was the use of the ICF’s Core sets for specific conditions, which contains a list of categories describing the most salient aspects of disability related to these. In this case, some of the components belonging to body functions (i.e. muscle power), and activities and participation (e.g. walking, eating, dressing) of the Core Set defined for stroke [124], could be assessed using wearable sensors.

The ICF seeks to provide comparable/replicable statistics of disability as a whole. The ICF’s performance and capacity qualifiers describe activities of daily living in natural environments and execution of specific tasks, respectively, which correspond to the “Diagnostics” and the “Evaluation of Recovery/Adaptation” dimensions described before for each sensor type. The “Extended Training” dimension addresses some social factors like degree of independence and integration to society by allowing patients to continue recovering after leaving the rehabilitation facility.

Baets and colleagues [125] reviewed the literature on shoulder assessment by means of IMUs, in the context of the ICF. They found that even though some measured aspects were repeatable and useful in this context, more work is needed to generate clinically meaningful, repeatable information. Standardizing measurements to characterize performance and capacity qualifiers, as described by the ICF, will also allow leveraging these datasets for the application of more complex analyses requiring larger amounts of data (e.g. neural networks).

#### Economic impact of stroke and potential benefits from wearable devices

The European Union spends €45 billion on treating stroke patients every year, with 44% of these costs spent on direct health care, 22% related to productivity losses and 35% on informal care of patients [126]. Care after stroke depends on how involved institutions (governments, healthcare centers, insurance companies, etc.) manage their resources [127], which influences the length of stay in the hospital and the extension of therapeutic care [128]. For instance in the United States, “Medicare” [129] has strict rules for the provision of intensive inpatient rehabilitation therapies (i.e. at least 3 h per day, 5 to 6 days per week), with an average length of stay of 15 days, at which point 70% of patients are sent home [130]. This percentage goes up to 90% after 3 months, and if patients have not recovered enough to be cared for at home by then, they will either receive more restricted healthcare coverage from state-based payers (e.g. “Medicaid”) or be sent to nursing homes where they will receive limited rehabilitation [130].

A study in Switzerland revealed that 37% of direct health care costs after stroke correspond to rehabilitation at the clinic [131]. Using systems like Hocoma’s “ArmeoSenso” [117] could allow patients to train in groups, which besides allowing therapists to tend to more people simultaneously, could bring enhanced effects of rehabilitation (e.g., [132]), rendering it more cost-effective. Motor assessments could be made more agile through wearable sensors, and patients could do it without a therapist being present (e.g. at home).

Results from meta-analyses have shown that early planned and coordinated hospital discharge combined with home-based rehabilitation yields better results, and home-based rehabilitation was found to be superior to center-based, as measured by the Bartel Index 6 months after stroke [133]. Healthcare coverage of home-based services can limit the length of therapy provided, but the use of wearable sensors for home-based therapy could grant access to these enhanced benefits while keeping costs low. Extended recovery resulting from home-based rehabilitative training (discussed in the next sub-section) could also increase the level of independence in patients, which would decrease costs related to productivity losses and informal care.

### Home-based self-application of rehabilitative training

Evidence of enhanced recovery related to more extensive training has been found in stroke patients [12], but high costs inherent to provided care, such as patient transportation or the therapy itself (i.e. therapists’ salary, rehabilitation site, etc.), often limit the therapies’ duration and frequency. On the other hand, training in more familiar environments, such as at home, improves the effects of training [134]. Training transfer to different environments, in general, is highly reduced [135], which is why training tasks should resemble activities of daily life, and take place at locations where they would occur on a daily basis.

Unsupervised, home-based rehabilitative training has the potential to largely improve outcome of rehabilitation in patients [136, 137]. Home-based training offers many advantages, but reducing contact between trainers and beneficiaries could impact motivation and engagement, which play a major role in recovery [5]. Therapists’ expertise would still be necessary to determine and adjust therapies, as well as to follow-up on training and rehabilitation progress, but contact between therapists and those under their care could be less frequent. This complicates the assessment of training quality and progress evaluation over shorter periods (daily, weekly), which might impact on motivation, planning of the intervention and personalized adaption of the treatment strategy [134]. Careful consideration of these potential threats is paramount to provide effective rehabilitation at home. Burridge and colleagues [138] discuss the effectiveness of some home-based rehabilitation systems and show that this approach is feasible and has the potential to improve motor function by training daily at home. They also present a new system (the “M-Mark”), which will allow patients to train at home under different circumstances of daily life (e.g. placing objects on a kitchen shelf) while being tracked by IMUs and mechanomyography.

### Practical considerations

There are many aspects to look into for home-based rehabilitative training and its corresponding assessments and measurements. First, training must be thoroughly and carefully explained to patients and, when applicable, to their caregivers. An option is to provide center/lab-based training for a short amount of time and then instruct patients to train at home [139]. Further, provided equipment must be as simple to use as possible to reduce chance of making mistakes and ensure training adhesion. An example of how possible mistakes can be reduced in a home-based environment can be found in the work of Durfee et al. [58], like blocking elements not useful to users (e.g. parts of the keyboard).

Another important aspect to consider is data logging. One option is to keep all data on the devices and extract it once the participants give the devices back at the end of their study contribution [31]. Nevertheless, this presents a risk with longer studies, as devices are lent for longer periods, and any accident damaging the device would result in loss of all previously gathered data. An alternative would be to relay the data to a protected server [61]. This could be challenging whenever participants’ homes are located in relatively isolated areas, with poor internet connection. Mobile broadband modules could solve this issue, although constraints from telecommunications companies providing the service still exist. Ultimately, it is most likely best to store data both on the devices and on a server, in a redundant manner.

Even though home-based training offers beneficial possibilities in terms of high-intensity training, other aspects, such as motivation derived from human interaction [5] might be lacking. For this reason, taking advantage of virtual conference tools (e.g. “Skype” [140]) could allow therapists to provide feedback and motivate patients, as well as to acquire feedback. A recent report by Maceira-Elvira and colleagues [141] discusses some of the challenges and important aspects to take into account in home-based training. The report highlights the importance of remote assistance and proper instructions provided to users, as well as technical assistance around the clock. Another report by Van de Winckel and colleagues [142] provides valuable information about the (generally positive) opinion of six patients enrolled on remotely-monitored home-based training.

## Conclusion

Stroke rehabilitation is an iterative process involving impairment assessment, recovery prognosis, therapy definition, rehabilitative training and monitoring of functional changes. Conventional assessments of motor function face limitations due to several factors, resulting in biased predictions of recovery, which prevent an adequate assignment of treatment for patients. Furthermore, limited resources at rehabilitation centers and clinics prevent patients from receiving intensive treatment and extensive attention, frequently reducing the degree up to which they recover. Wearable sensors show promise resolving at least some of these problems. Regular assessments complemented with this technology can reduce bias in measurements and estimations, as well as reduce assessment time for therapists. Short-term rehabilitative training, offered during the first 6 months after stroke, could be prolonged by offering home-based therapies, designed and monitored remotely by therapists, allowing patients to train in a familiar environment. Among the wide array of sensors available, inertial measurement units (IMUs) and electromyography (EMG) offer the best balance between unobtrusiveness, robustness, ease of use and data quality. An optimal solution comprising both sensor types is still lacking in the market, but the collection of studies presented in this review indicate that this might be the most promising way to go.

## Data Availability

Not applicable.

## Abbreviations

(s)EMG: (surface) Electromyography
(UE)FM: (Upper extremity) Fugl-Meyer
ARAT: Action research arm test
AUC: Area under the curve
BLE: Bluetooth Low-Energy
CE: Conductive elastomer
CPD: Coherent point drift
DTW: Dynamic time warping
ELM: Extreme learning machines
FAS: Functional ability score
FMA: Fugl-Meyer Assessment
ICF: International Classification of Functioning, Disability and Health
IMU(s): Inertial measurement unit(s)
KNN: K-nearest neighbor
LASSO: Least absolute shrinkage and selection operator
LDA: Linear discriminant analysis
LED: Light-emitting diode
MEMS: Microelectromechanical systems
MEP: Motor-Evoked Potential
NMF: Non-negative matrix factorization
PCA: Principal component analysis
RBF: Radial basis functions
RMSE: Root mean square error
ROC: Receiver operating characteristic
ROM: Range of motion
SVM: Support vector machines
TMS: Transcranial Magnetic Stimulation
WMFT: Wolf motor function test
